# Glutamine Metabolism Is Required for Alveolar Regeneration during Lung Injury

**DOI:** 10.3390/biom12050728

**Published:** 2022-05-22

**Authors:** Sisi Wang, Xue Li, Qingwen Ma, Qi Wang, Junping Wu, Hongzhi Yu, Kuan Li, Yu Li, Jianhai Wang, Qiuyang Zhang, Youwei Wang, Qi Wu, Huaiyong Chen

**Affiliations:** 1 Department of Basic Medicine, Haihe Clinical School, Tianjin Medical University, Tianjin 300350, China; wangsisi@tmu.edu.cn (S.W.); qingwen20201216@163.com (Q.M.); 2 Department of Basic Medicine, Haihe Hospital, Tianjin University, Tianjin 300350, China; lixue1986fly@126.com (X.L.); wangdake1992@163.com (Q.W.); kuan_li1990@126.com (K.L.); 18649019165@189.cn (Y.L.); jianhai.wang@outlook.com (J.W.); qy_zhang0923@126.com (Q.Z.); 3 Department of Tuberculosis, Haihe Hospital, Tianjin University, Tianjin 300350, China; wujp0618@126.com (J.W.); 13512205663@163.com (H.Y.); 4 Academy of Medical Engineering and Translational Medicine, Tianjin University, Tianjin 300072, China; 5 Key Research Laboratory for Infectious Disease Prevention for State Administration of Traditional Chinese Medicine, Tianjin Institute of Respiratory Diseases, Tianjin 300350, China; 6 Tianjin Key Laboratory of Lung Regenerative Medicine, Tianjin 300350, China

**Keywords:** glutamine metabolism, idiopathic pulmonary fibrosis, alveolar progenitor cells, lung regeneration, omics

## Abstract

(1) Background: Abnormal repair after alveolar epithelial injury drives the progression of idiopathic pulmonary fibrosis (IPF). The maintenance of epithelial integrity is based on the self-renewal and differentiation of alveolar type 2 (AT2) cells, which require sufficient energy. However, the role of glutamine metabolism in the maintenance of the alveolar epithelium remains unclear. In this study, we investigated the role of glutamine metabolism in AT2 cells of patients with IPF and in mice with bleomycin-induced fibrosis. (2) Methods: Single-cell RNA sequencing (scRNA-seq), transcriptome, and metabolomics analyses were conducted to investigate the changes in the glutamine metabolic pathway during pulmonary fibrosis. Metabolic inhibitors were used to stimulate AT2 cells to block glutamine metabolism. Regeneration of AT2 cells was detected using bleomycin-induced mouse lung fibrosis and organoid models. (3) Results: Single-cell analysis showed that the expression levels of catalytic enzymes responsible for glutamine catabolism were downregulated (*p* < 0.001) in AT2 cells of patients with IPF, suggesting the accumulation of unusable glutamine. Combined analysis of the transcriptome (*p* < 0.05) and metabolome (*p* < 0.001) revealed similar changes in glutamine metabolism in bleomycin-induced pulmonary fibrosis in mice. Mechanistically, inhibition of the key enzymes involved in glucose metabolism, glutaminase-1 (GLS1) and glutamic-pyruvate transaminase-2 (GPT2) leads to reduced proliferation (*p* < 0.01) and differentiation (*p* < 0.01) of AT2 cells. (4) Conclusions: Glutamine metabolism is required for alveolar epithelial regeneration during lung injury.

## 1. Introduction

Functional regeneration of damaged tissues is necessary to restore homeostasis in normal organs. A representative example is that satellite cells in skeletal muscle are activated in response to injury and differentiate into myoblasts to rebuild muscle fibers [1]. Active regeneration of the intestine is also inseparable from the resident intestinal stem cells (ISCs) located at the base of the crypt, and the regenerative ability emerges from all three germ layers through coordinated plasticity [2]. Similarly, once lung injury occurs, endogenous region-specific stem/progenitor cells re-enter the cell cycle and divide or differentiate at an accelerated rate to facilitate repair [3,4]. As a lung resident progenitor cell population, alveolar type 2 (AT2) cells maintain an abundance of alveolar epithelial cells in the distal lung, and it has been shown that a variety of signaling pathways, including the Wnt/β-catenin and TGF-β signaling pathways, are involved in AT2 cell proliferation and differentiation during alveolar regeneration [5]. Recent evidence suggests that the inability of the epithelium to repair, regenerate, and remodel effectively can be attributed to aging, mechanical tension, microenvironment injury, and reactive oxygen species [6,7,8,9]. These factors are largely consistent with the reported epidemiological risk factors for idiopathic pulmonary fibrosis (IPF), which include cigarette smoking, environmental exposures, microbial pathogens, and genetic risk factors [10]. These factors disrupt cell metabolism, resulting in the ongoing injury to reach the alveolar epithelium and subsequent disease. Therefore, a well-established and explainable metabolic mechanism for alveolar cell fate needs to be investigated.

IPF has an unknown cause, limited therapeutic effect and poor prognosis, and is characterized by persistent acute lung injury followed by scar formation and end-stage lung disease. Multiple forms of lung injury, including humoral autoimmunity, endothelial dysfunction, granuloma formation, or alveolar macrophage activation may contribute to the progression of fibrotic phenotypes [11]. Among the above, it has been gradually recognized that the vicious circle of abnormal epithelial injury and post-injury repair is a key mechanism underlying the pathogenesis of progressive pulmonary fibrosis [12,13]. The mutation rates of alveolar cell-related genes, *SFTPC* and *ABCA3*, in patients with IPF have been found to be higher than those in healthy controls, and the imaging manifestations of patients with these gene mutations are also significantly different from those corresponding to healthy controls [14]. As alveolar epithelial progenitors, AT2 cells can proliferate to replace the lost AT2 cells and differentiate into alveolar type 1 (AT1) cells during lung injury [4,15]. Energy consumption is essential for regulating cellular functions. Accumulating evidence suggests that the activation of progenitor cell regeneration is widely influenced by metabolic programs [16,17]. Previously, we observed increased glucose metabolism and reduced lipid metabolism in AT2 cells in bleomycin-induced mouse lung fibrosis, and inhibition of glycolysis or pentose phosphate pathway resulted in restricted AT2 cell proliferation and accelerated pulmonary fibrosis progression [18]. This may be due to the production of large amounts of adenosine triphosphate (ATP) from glycolysis and aerobic oxidation during glucose metabolism, which is beneficial for lung epithelial regeneration. Fatty acid oxidation slows the resolution of pulmonary fibrosis and delays alveolar repair by abolishing self-renewal and differentiation of AT2 cells and bronchioalveolar stem cells (BASCs) [19]. In either case, the AT2 cells reprogram their metabolism to support regeneration.

Glutamine is another important metabolic fuel for cells, helping rapidly proliferating cells meet their increasing demand for ATP and biosynthetic precursors [20,21,22]. Glutamine enters cells via amino acid transporters and is converted to glutamate in the mitochondria via deamination catalyzed by glutaminase-1 (GLS1). Glutamate is converted to the tricarboxylic acid cycle (TCA cycle) intermediate α-ketoglutaric acid (α-KG) by glutamic-pyruvic transaminase-2 (GPT2) or glutamic-oxaloacetic transaminase (GOT) [23]. GLS1 activity and glutamine metabolism are necessary for the proliferation and differentiation of skeletal stem cells [24]. Glutamine can promote the proliferation of small ISCs and differentiate into goblet cells, Paneth cells, and intestinal endocrine cells [25]. Increased glutamine has been observed in lung cancer tissue and the elevated glutamine promote neoplastic transformation and progression [26]. Moreover, glutamine metabolism promotes the activation of several signaling pathways, such as the mammalian target of rapamycin (mTOR) and mitogen-activated protein kinase (MAPK) pathways, to support tumor cell growth and proliferation [27]. The glutamine metabolite gamma-aminobutyric acid (GABA) promotes mucin secretion by goblet cells during asthma progression [28]. Lung myofibroblasts demonstrate markedly augmented glutaminolysis mediated by elevated GLS1 in transforming growth factor beta 1 (TGFβ1)-stimulated fibroblasts [29]. However, the role of glutamine metabolism in alveolar progenitor cells during lung epithelial regeneration in fibrosis remains unclear.

In this study, we investigated the expression of the catalytic enzymes that are responsible for glutamine catabolism in AT2 cells from patients with IPF via single-cell RNA sequencing (scRNA-seq) analysis. Further, we also investigated the changes in glutamine metabolism in AT2 cells from mice with bleomycin-induced lung fibrosis via transcriptomics and metabolomics, and combined with the use of an organoid model, we revealed the role of glutamine metabolism in the proliferation and differentiation of AT2 cells using different inhibitors in vitro. Our results support the notion that glutamine metabolism is instrumental in driving the metabolic switch in alveolar progenitor cells to meet energy requirements for epithelial homeostasis and regeneration.

## 2. Materials and Methods

### 2.1. Mice

C57BL/6J mice were purchased from Beijing Huafukang Bioscience Co., Inc. (Beijing, China). All experimental mice were housed in a specific pathogen-free facility at the Tianjin Haihe Hospital. All mice were exposed to a 12-h light/dark cycle and had free access to food and water. Assigned to control or treatment groups, age-matched mice between 8–12 weeks were used for organoid cultures in vitro and bleomycin-induced lung injury experiments in vivo. All animal experiments were performed in accordance with guidelines approved by the Tianjin Haihe Hospital Animal Care and Use Committee (2020HHKT-022).

### 2.2. Bleomycin-Induced Lung Injury

The experimental mice were anesthetized by an intraperitoneal injection of 7.5% chloral hydrate, and bleomycin (2 U/kg) or phosphate-buffered saline (PBS) was intratracheally injected as we previously described [18]. Mouse lung tissues were harvested on days 14 after bleomycin treatment for histological staining or flow cytometry analysis.

### 2.3. Mouse Lung Dissociation and Flow Cytometry

As mentioned previously [18], mouse lung single-cell suspension was obtained by digestion of lung tissues with 4 U/mL elastase and DNase I at 37 °C. Then, isolated cells were resuspended in Hank’s balanced saline solution (HBSS) with 2% FBS, 100 IU/mL penicillin, 100 μg/mL streptomycin, and 10 mM HEPES and incubated with the following primary antibodies: CD31-biotin, CD34-biotin, CD45-biotin, CD24-PE, EpCAM-PE-Cy7, and Sca-1-APC. The secondary antibody was streptavidin. 7-Amino-actinomycin D was used to identify dead cells. Flow cytometry was performed with a FACS Aria III sorter (BD Biosciences, San Jose, CA, USA) and CD31^−^CD34^−^CD45^−^EpCAM^+^CD24-Sca-1^−^ AT2 cells were sorted for organoid culture or RNA analyses.

### 2.4. Organoid Culture

Flow-sorted mouse AT2 cells (2 × 10^4^ cells per well) were cultured with mouse lung fibroblast MLg (2 × 10^5^ cells per well) (CCL-206, ATCC) and Matrigel (BD Pharmingen, San Diego, CA, USA) added to 24-well Transwell filter inserts (Greiner BioOne, Kremsmünster, Austria) as described previously [18]. Organoid cultures were maintained at 37 °C incubator containing 5% CO_2_ and fresh medium was renewed every other day. The colonies were observed using an Olympus IX37 inverted fluorescence microscope. Colonies with diameters ≥ 50 μm were counted, and colony-forming efficiency (CFE) was determined on day 10 after inoculation. To inhibit glutaminase in the glutamine metabolic pathway, a glutamine analog, 6-diazo-5-oxo-L-norleucine (DON, Selleckchem, Houston, TX, USA), was added to the cell culture medium. To inhibit GOT and GPT2 in the glutamine metabolic pathway, aminooxyacetic acid hemihydrochloride (AOA, Selleckchem), an inhibitor of aminobutyrate aminotransferase, was added to the cell culture medium.

### 2.5. RNA Extraction and Real-Time PCR

RNA was extracted from AT2 cells and organoid cultures using TRIzol reagent (Invitrogen) following the manufacturer’s instructions. A total of 0.2 μg of total RNA was used for reverse transcription. SYBR Green Supermix and a Light Cycler 96 real-time PCR system were used for quantitative real-time PCR. The conditions of RT-PCR amplification were: 95 °C for 120 s, 40 cycles of 95 °C for 10 s, 60 °C for 20 s, and 72 °C for 20 s. Gene expression level was measured relative to the endogenous reference gene, mouse β-actin. The primer sequences were as follows: β-actin-F: 5′-GGCCAACCGTGAAAAGATGA-3′; β-actin-R: 5′-CAGCCTGGATGGCTACGTACA-3′; Gls1-F: 5′-TTCGCCCTCGGAGATCCTAC-3′; Gls1-R: 5′-CCAAGCTAGGTAACAGACCCT-3′; Gpt2-F: 5′-TCCAGGCTTCAAGGAATGGAC-3′; Gpt2-R: 5′-GGACTGCATACTCCACCCT-3′; T1α-F: 5′-TGCTACTGGAGGGCTTAATGA-3′; T1α-R: 5′-TGCTGAGGTGGACAGTTCCT-3′.

### 2.6. Detection of Glutamate and α-Ketoglutarate

Glutamate and α-ketoglutarate were quantitatively detected using glutamate and α-ketoglutarate content determination kits (Jining Industry, Shanghai, China) following the manufacturer’s instructions.

### 2.7. Determination of Hydroxyproline Content

Hydroxyproline content was quantitatively determined using a hydroxyproline assay kit (Jiancheng Industry, Nanjing, China) following the manufacturer’s instructions.

### 2.8. Histopathological Staining of Mouse Lung Tissue

Mice were euthanized and lung tissues were isolated on day 14 after bleomycin treatment for histological staining. H&E staining was performed as described previously [18]. Immunofluorescence staining was performed as previously described [30]. In brief, 5 μm lung slices were deparaffinized, rehydrated and incubated with rabbit polyclonal anti-proSPC (Millipore, AB3786, 1:200 dilution) and hamster polyclonal anti-Pdpn (nvitrogen, 1:200 dilution). Lung sections were then incubated with fluorochrome-conjugated secondary antibodies (1:200; Invitrogen) at room temperature for 90 min. Thereafter, sections were mounted with Fluoromount G containing 4-6-diamidino-2-phenylindole (DAPI) for 30 min. The stained sections were observed under an IX73 inverted fluorescent microscope (Olympus, Tokyo, Japan).

### 2.9. AT2 Cell Viability Assay

AT2 cells (2 × 10^4^ cells/well) were seeded into a 96-well plate (Labgic, Beijing, China). Thereafter, inhibitors with different concentration gradients were added into the wells followed by culturing at 37 °C for 24 h. Thereafter, 3-(4,5-dimethylthiazol-2-yl)-5-(3-carboxymethoxyphenyl)-2-(4-sulfophenyl)-2H-tetrazolium (MTS) was added directly to the culture well and the effect of each compound on the viability of AT2 cells was evaluated at a wavelength of 490 nm using a Spectrophotometer (Multiskan Go, Thermal Fisher Scientific, Waltham, MA, USA).

### 2.10. Single-Cell RNA-seq Analysis

Two datasets (GSE135893 and GSE136831) were obtained from the Gene Expression Omnibus (GEO) database of the National Center for Biotechnology Information (NCBI). Single-cell RNA-seq of IPF and control patients was performed using a 10× chromium platform. The raw expression matrix and patient metadata of the two GEO series were downloaded. Data processing and visualization were performed using R software (version 4.1.2, Cambridge, MA, USA). The batch effects of the inner samples and outer series were removed using the harmony package (version 0.1.0). Multiplets of raw data were detected using the DoubletFinder package (version 2.0.3) and removed before analysis. Downstream analysis was performed using the Seurat package (version 4.0.6). For quality control, genes expressed in less than three cells and cells expressing less than 200 genes were excluded from the expression matrix of each sample; cells containing more than 20% of the reads derived from mitochondria were also removed. Then, all the individual samples were merged, and the standard workflow (normalization, dimensionality reduction, clustering, and visualization) of the Seurat package was performed with default parameters. The main and fine cell types were annotated using canonical markers [31,32]. Differentially expressed genes were detected using the FindMarkers function of the Seurat package.

### 2.11. Transcriptomic and Metabolomic Analyses

Transcriptome sequencing and metabolomic analysis of AT2 cells is described in our previous study [18]. The RNA-seq data (GSE143212) can be obtained from the series matrix files downloaded from the GEO database. Significant differentially expressed genes were screened by *p* < 0.05 and fold-change (FC) ≥ 2 to compare the changes in genes related to glutamine metabolism. The overall distribution of the differences between the two groups is reflected in a volcano map. Unsupervised hierarchical clustering of differentially expressed genes was performed and the results are reflected in a heat map. We analyzed the enriched Kyoto Encyclopedia of Genes and Genomes (KEGG) pathway using MetaboAnalyst 4.0 webservice (http://www.metaboanalyst.ca, accessed on 10 March 2021). Similar to the transcriptome analysis, the metabolomics analysis has been described in detail in a previous study [18]. Here, we reanalyzed the data by focusing on the substances associated with glutamine metabolism. The total ion flow chromatograms of all the samples were visualized to ensure a strong instrumental analysis signal, large peak capacity, and good retention time reproducibility. Variable importance in the projection (VIP) was used to rank the overall contribution of each variable to the orthogonal partial least squares discriminant analysis (OPLS-DA) model, and variables with VIP > 1.0 were considered as differential variables. We identified metabolites with both multivariate and univariate statistical significance (VIP > 1.0, *p* < 0.05) as differential metabolites.

### 2.12. Statistical Analysis

Regarding scRNA-seq data, the *p* values corresponding to differentially expressed gene analysis were determined by performing a two-sided Wilcoxon rank sum test using the “FindMarkers” function of the Seurat package in R. For bleomycin-induced lung injury experiments and organoid culture experiments, the data obtained were presented as the mean ± standard error of the mean (SEM) and the Student’s *t*-test was performed to determine statistically significant differences, with statistical significance set at *p* < 0.05 (* *p* < 0.05; ** *p* < 0.01; *** *p* < 0.001).

## 3. Results

### 3.1. Alveolar Progenitor Cells in Patients with IPF Exhibit Downregulated Glutamine Catabolism

**Figure 1:**
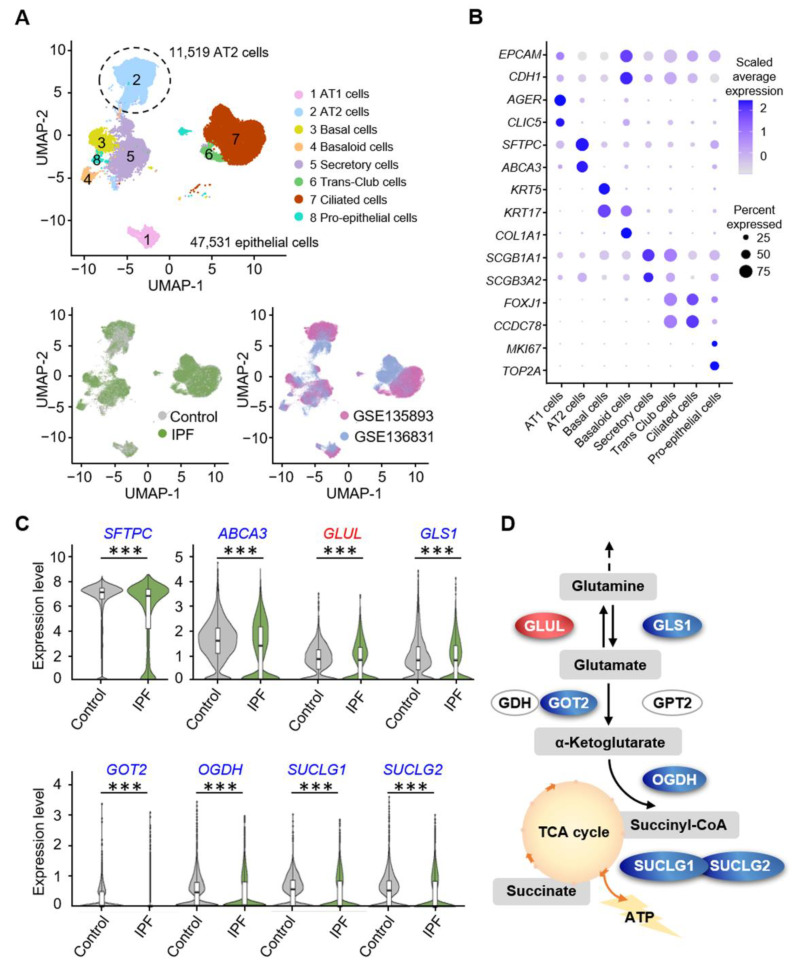
Glutamine metabolism is downregulated in AT2 cells of patients with IPF. (**A**) Uniform Manifold Approximation and Projection (UMAP) graphical representation of 47,531 epithelial cells from 44 IPF and 38 control lungs (top), disease status (bottom left), and subjects (bottom right). Each dot represents a single cell and cells are labeled as one of eight discrete cell varieties, with AT2 cells labeled by the number 2. (**B**) Dotplot of the canonical marker gene expression in eight epithelial cell types. Node size is proportional to the percentage of cells in the cluster expressing a gene. Node color is proportional to the average expression level of the gene in the cluster. (**C**) Violin plots show gene expression in AT2 cells from the samples in 1A, and the upregulated (red) gene *GLUL* and the downregulated (blue) genes *SFTPC*, *ABCA3*, *GLS1*, *GOT2*, *OGDH*, *SUCLG1*, and *SUCLG2* are depicted. (**D**) Glutamine metabolism pathway diagram shows the genetic alterations that regulate the related enzymes, according to the integrated analysis of scRNA-seq data from healthy donors or patients with IPF. Upregulated and downregulated genes are marked in red and blue, respectively. *** *p* < 0.001.

A fibrotic alveolar decline in metabolism can lead to irreversible and progressive respiratory insufficiency [33]. To better understand the role of glutamine metabolism in lung injury, we retrieved and integrated the single-cell data of 44 IPF patients and 38 healthy controls obtained from the GSE135893 [31] and GSE136831 [34] datasets. We comprehensively analyzed the raw data, removed low-quality cells, and then performed the standard workflow of the Seurat [35,36] package in R software. Compelling evidence suggested that IPF represents an epithelial-driven disorder [36,37]; hence, we conducted a more focused study of epithelial cells (EPCAM+, CDH1+). We selected a total 47,531 epithelial cells for further analysis, and clustered them into eight cell types or states according to canonical lineage-defining cell markers, including three alveolar clusters (AT1 cells, AT2 cells, and Basaloid cells), four airway clusters (Basal cells, Secretory cells, Trans-Club cells, Ciliated cells), and one proliferating cluster (Pro-epithelial cells) (Figure 1A,B). Further, to reduce batch effects on the clustering and perform differentially expressed gene analysis, we established an algorithm using the harmony package [38,39]. Thus, cells from different clinical groups and GEO series were evenly distributed among all the eight cell varieties in the Uniform Manifold Approximation and Projection (UMAP) plot. Thus, no obvious batch-associated clusters were observed, implying that the batch effects between samples were sufficiently removed (bottom of Figure 1A). We focused on the differentially expressed genes related to glutamine metabolism in 11,519 AT2 cells. The glutamine cell membrane exchanger comprises alanine-serine-cysteine transporter 2 (ASCT2) and large neutral amino acid transporter 1 (LAT1) with the ancillary subunit CD98. Genes for all three transporters were downregulated simultaneously, whereas no significant changes were observed in glutamine metabolism in the cytoplasm. First, we observed that the expression of AT2 cell markers, Surfactant Protein C (SFTPC) (*p* = 4.27 × 10^−71^) and Adenosine triphosphate binding cassette subfamily A member 3 (ABCA3) (*p* = 8.87 × 10^−38^) were downregulated. Next, we focused on glutamine metabolism in mitochondria. GLS1 converts glutamine γ-nitrogen to produce glutamate and free ammonia, and the rate-limiting enzyme GLS1 in the glutamine replenishment pathway was downregulated (*p* = 3.03 × 10^−49^). Simultaneously, the conversion of glutamate to α-ketoglutarate regulates the entry of glutamine-derived carbon into the TCA cycle by decreasing glutamic-oxaloacetic transaminase 2 (GOT2) and 2-oxoglutarate dehydrogenase (OGDH) (*p* = 1.47 × 10^−40^ and *p* = 8.64 × 10^−56^, respectively). In particular, decreased expression of the succinate-CoA ligase (SUCLG) complex, the key enzyme in the rate-limiting step of the TCA cycle, may directly affect AT2 productivity (SUCLG1, *p* = 9.69 × 10^−106^; SUCLG1, *p* = 1.47 × 10^−89^). At the same time, glutamate ammonia ligase (GLUL), which regulates glutamine synthesis, was upregulated (*p* = 5.56 × 10^−30^), resulting in the accumulation of glutamine (Table S1 and Figure 1C). These effects may directly mediate the decrease in intracellular glutamate, α-ketoglutarate, and ATP levels (Figure 1D). Collectively, these observations indicate that there is reduced glutamine exchange with the outside and downregulated catabolism in the mitochondria of AT2 cells from patients with IPF.

### 3.2. Glutamine Metabolic Pathway Is Inhibited in AT2 Cells of Bleomycin-Induced Fibrosis in Mice

To further study the regulation of glutamine metabolism on alveolar epithelial repair in IPF, we induced pulmonary fibrosis with bleomycin in mice. Pathological staining of the mouse lung tissue 14 days after bleomycin administration showed a distorted alveolar structure (Figure S1A). Immunofluorescence staining and flow cytometry analysis revealed that the abundance of AT2 and AT1 cells in the alveolar epithelium decreased significantly after bleomycin injury (Figure S1B–D). The hydroxyproline content of the lungs of mice at 14 days after bleomycin administration was significantly higher than that corresponding to the control mice (Figure S1E). Similarly, inhibition of glutamine metabolism in AT2 cells was observed during bleomycin-induced lung fibrosis in mice. To confirm this, we performed organoid culture of AT2 cells (Figure S1F–H). The colony formation efficiency (CFE) of the surviving AT2 cells from bleomycin injury was lower than that of AT2 cells from PBS-treated mice. These results reveal that AT2 cells may lack the capacity to self-renew and differentiate into AT1 cells during bleomycin-induced injury.

**Figure 2:**
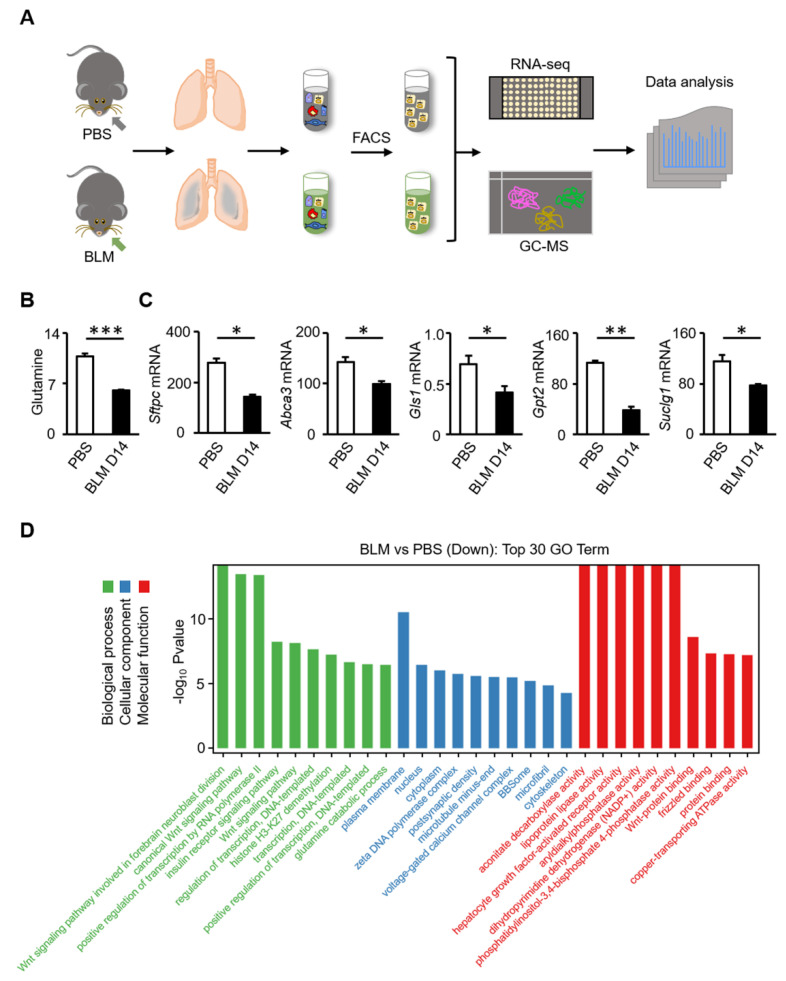
Glutamine metabolic pathway is inhibited in AT2 cells of bleomycin-induced fibrosis in mice. (**A**) Overview of experimental design. AT2 cells from mice treated with PBS or bleomycin for 14 days were isolated by FACS to generate a single-cell suspension for both scRNA-seq and metabolomics analyses. (**B**) Expression of glutamine metabolite in AT2 cells was downregulated after bleomycin injury. (**C**) Expression levels of *Sftpc*, *Abca3*, *Gls1*, *Gpt2* and *Suclg1* genes in AT2 cells were decreased after bleomycin injury. (**D**) Top 30 most enriched Gene Ontology (GO) terms of downregulated differentially expressed genes (DEGs). * *p* < 0.05, ** *p* < 0.01, *** *p* < 0.001.

To investigate the regulation of glutamine metabolism on the regeneration of AT2 cells during lung injury, RNA-seq and metabolic profiling were performed with AT2 cells from mice treated with bleomycin (Figure 2A). The volcano map shows the distribution of differences in gene expression levels between the bleomycin and PBS groups (*p* < 0.05) (Figure S2A). Differential expression analysis revealed that there were 1685 differentially expressed genes between the two groups (FDR-adjusted *p* < 0.05), of which 928 were upregulated and 756 were downregulated (Figure S2B). Visualization of the total ion current (TIC) showed that instrumental analysis of all samples had a strong signal, large peak capacity, and good retention time reproducibility (Figure S2C). Multi-dimensional and single-dimensional analyses were used to screen the differential metabolites between the groups. The OPLS-DA score chart separated the bleomycin and PBS groups (Figure S2D), which indicated marked disease-specific transcriptomic differences. According to the metabolic profiling, the levels of glutamine metabolites decreased (*p* < 0.001) (Figure 2B). Transcriptomic analysis showed that the expression levels of Gls1 (*p* = 0.041) and Gpt2 (*p* = 0.001) were downregulated (Figure 2C). The same trend was evident for Suclg1 (*p* = 0.046), a subunit gene of succinyl-CoA ligase (SUCL) in AT2 cells. The expression of AT2 markers was also reduced (Sftpc, *p* = 0.010; Abca3, *p* = 0.043) (Figure 2C). The top 30 most enriched Gene Ontology (GO) terms of downregulated differentially expressed genes (DEGs) are shown in Figure 2D. There are three GO categories, namely, biological process (BP), cellular component (CC), and molecular function (MF), which describe the possible molecular functions, cellular environment, and biological processes of gene products, respectively. Specifically, the glutamine catabolic process and the canonical Wnt signaling pathway were identified as the major biological processes, and, as a major downstream effector of the classical Wnt signaling pathway, activated β-catenin regulates the expression of glutamine synthase. Further, genes regulating dihydro-pyrimidine dehydrogenase (NADP+) and ATPase activity were also downregulated.

Combined with the above results, the scRNA analysis of IPF and the transcriptomic/metabolomic analysis of bleomycin-induced fibrosis mice similarly revealed downregulated glutamine metabolism entering the mitochondria, which may be involved in the pathogenesis of pulmonary fibrosis (Figure S3). There is little evidence of significant changes in glutamine inflow, outflow, and cytoplasmic pathways during bleomycin-induced lung fibrosis in mice.

### 3.3. Inhibition of GLS1 and GPT2 in AT2 Cells In Vitro Leads to Decreased Glutamine Metabolism

**Figure 3:**
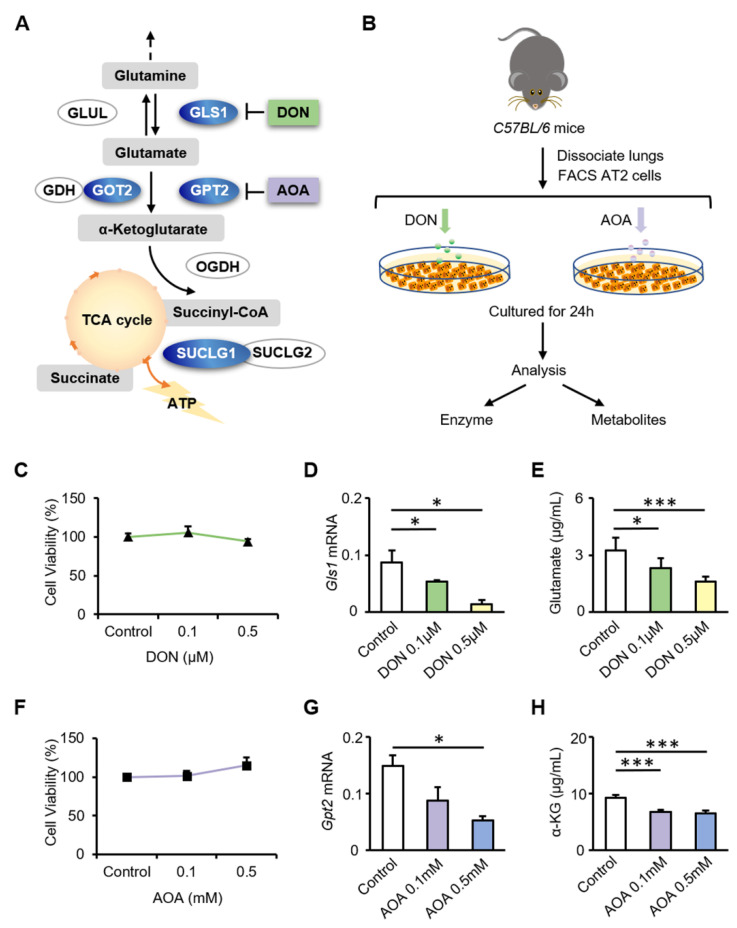
Inhibition of GLS1 and GPT2 in AT2 cells in vitro leads to decreased glutamine metabolism. (**A**) Schematic diagram showing alterations in enzymes associated with glutamine metabolism in AT2 cells. Enzymes with decreased expression are labeled in blue. DON and AOA are inhibitors of GLS1 and GPT2, respectively. (**B**) AT2 cells treated with glutamine metabolic inhibitors. (**C**) Viability of AT2 cells unaffected by DON at a concentration of 0.5 μM or lower (*n* = 3). (**D**,**E**) Gls1 mRNA expression and glutamate metabolite content in AT2 cells after 24 h of DON stimulation. (**F**) Viability of AT2 cells unaffected by AOA at a concentration of 0.5 mM or lower (*n* = 3). (**G**,**H**) Gpt2 mRNA expression and α-ketoglutarate metabolite content in AT2 cells after 24 h of AOA treatment. * *p* < 0.05, *** *p* < 0.001.

Glutamine is catalyzed by GLS1 to produce glutamate in the mitochondria. Glutamate is converted to α-KG by GPT2 or GOT2. α-KG enters the TCA cycle as an intermediate, accelerating cellular ATP production. DON inhibits the activity of GLS1. AOA, which is considered as an inhibitor of glutamate-dependent transaminases, was used to inhibit the glutamate-catalyzed reaction of α-KG that is mediated by GPT2 and GOT2 (Figure 3A). AT2 cells were treated with DON or AOA and analyzed 24 h later (Figure 3B). MTS was used to evaluate the cytotoxicity of DON and AOA. Based on the MTS analysis, the viability of AT2 cells was not affected by inhibitors at the tested concentrations (Figure 3C,F). Treatment of cells with DON resulted in a significant downregulation of Gls1 (0.1 μM DON, *p* = 0.036; 0.5 μM DON, *p* = 0.026) (Figure 3D), in parallel with a significant downregulation of glutamate (0.1 μM DON, *p* = 0.021; 0.5 μM DON, *p* < 0.001) (Figure 3E). Upon stimulation with AOA, AT2 cells showed downregulated expression of Gpt2 (0.1 mM AOA, *p* = 0.143; 0.5 mM AOA, *p* = 0.002) (Figure 3G), accompanied by a significant reduction in α-ketoglutarate (0.1 mM AOA, *p* < 0.001; 0.5 mM AOA, *p* < 0.001) (Figure 3H). These results indicate that AOA and DON effectively inhibit the activity of glutamine metabolic catalytic enzymes and reduce the generation of α-KG.

### 3.4. Inhibition of Glutamine Metabolism Reduces the Proliferation and Differentiation of AT2 Cells

**Figure 4:**
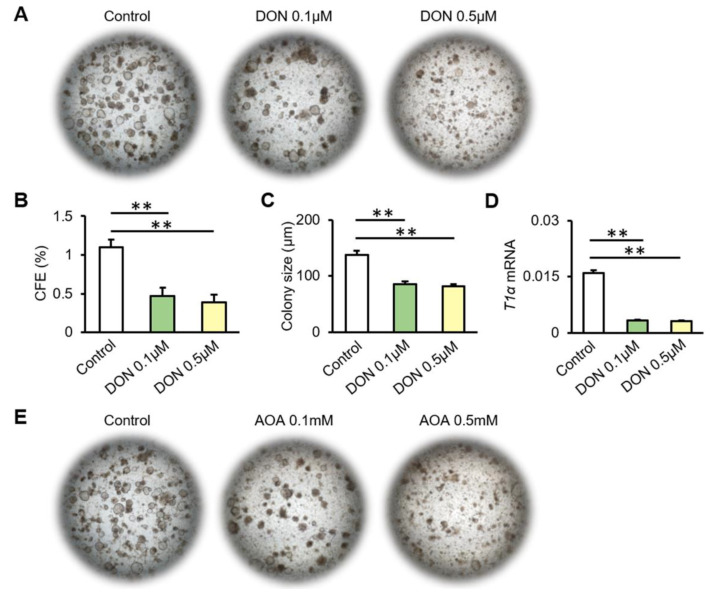

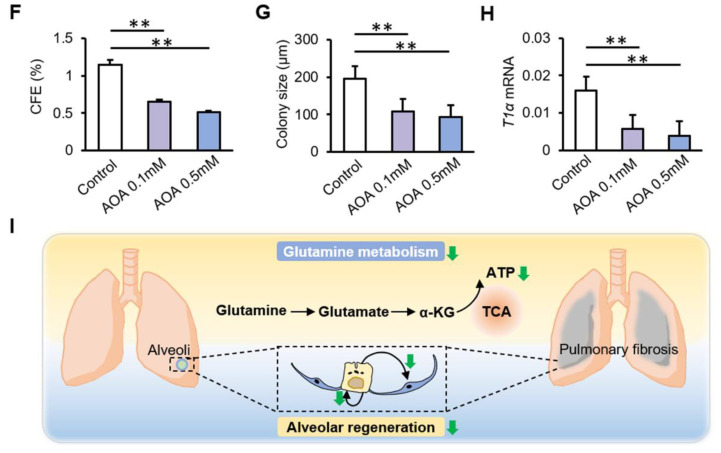
Inhibition of glutamine metabolism reduces the proliferation and differentiation of AT2 cells. (**A**) Representative micrographs of organoid cultures of mouse AT2 cells cultured with DON. (**B**,**C**) CFEs (**B**) and colony size (**C**) of organoid colonies. (**D**) T1α mRNA expression of organoid colonies after stimulation with DON. (**E**) Representative micrographs of organoid cultures of AT2 cells cultured with AOA. (**F**,**G**) CFEs (**F**) and colony size (**G**) of organoid colonies. (**H**) T1α mRNA expression of organoid colonies after stimulation with AOA. (**I**) Schematic diagram describing a possible contribution of glutamine metabolism to the proliferation and differentiation of alveolar progenitor cells. ** *p* < 0.01.

To investigate the regulation of glutamine metabolism in alveolar epithelium regeneration, we performed organoid cultures of AT2 cells in vitro. The colony-forming efficiency (CFE) (0.1 μM DON, *p* = 0.008; 0.5 μM DON, *p* = 0.006) and colony size (0.1 μM DON, *p* = 0.027; 0.5 μM DON, *p* = 0.019) were reduced in the presence of DON (Figure 4A–C). The expression levels of T1α (0.1 μM DON, *p* = 0.003; 0.5 μM DON, *p* = 0.001) decreased after DON treatment (Figure 4D). Likewise, AOA downregulated the CFE (0.1 mM AOA, *p* = 0.026; 0.5 mM AOA, *p* = 0.018) and organoid size (0.1 mM AOA, *p* = 0.021; 0.5 mM AOA, *p* = 0.020) of AT2 cultures, as well as T1α mRNA expression (0.1 mM AOA, *p* = 0.007; 0.5 mM AOA, *p* = 0.006) (Figure 4E–H). The differentiation potential of AT2 cells into AT1 cells was impaired. This suggests that it is difficult for damaged AT2 cells to maintain their abundance for further repair and self-renewal without normal glutamine metabolism. Previous studies have emphasized the important role of glutamine metabolism in epithelial cells. This study revealed a new regulation of glutamine metabolism in alveolar progenitor cells during lung fibrosis. Overall, these results show decreased glutamine metabolism in AT2 cells from both patients with IPF and mice with bleomycin-induced fibrosis. Inhibition of glutamine metabolism significantly reduced proliferation and differentiation of AT2 cells in vitro. This may result from the reduction in the glutamine metabolite α-KG, which enters the TCA cycle as an intermediate, resulting in the inhibition of ATP production, which is not conducive to rapid cell regeneration during lung injury (Figure 4I).

## 4. Discussion

Our study showed that the downregulated expression of the catalytic enzymes that are responsible for glutamine catabolism in AT2 cells from patients with IPF and mice with bleomycin-induced lung fibrosis. Mechanistically, the inhibition of the key metabolic enzymes in glutamine metabolism led to reduced AT2 cell proliferation and differentiation.

Increasingly optimized techniques and shareable data have accelerated the powerful exploration of cell biological functions at the single-cell level [40]. Single-cell RNA sequencing of 20 fibrotic lungs and 10 control lungs provided high-resolution insights into the complexity and plasticity of the distal lung epithelium [31]. A similar single-cell sequencing analysis of 32 versus 28 samples revealed the complexity and diversity of aberrant cellular populations in IPF [34]. In our study, the scRNA analysis showed that the expression of AT2 markers is reduced in samples of IPF patients. This might reflect that the AT2 cells were damaged and secreted a decreased level of surfactant proteins in IPF. There have been many studies on the effect of cellular energy metabolism on the progression of pulmonary fibrosis; however, the regulatory mechanism of glutamine metabolism in alveolar epithelial progenitor cells remains unclear. To explore the effect of glutamine metabolism on alveolar epithelial regeneration and repair, we comprehensively analyzed previously published IPF single-cell sequencing data. In terms of glutamine metabolism, we observed decreased expression of GLS1, GOT2, OGDH, and SUCLG in IPF patients, indicating downregulated glutamine metabolism in the mitochondria. The decline of genes involved in glutamine metabolism might correspond to a more general decline of mRNA in surviving AT2 cells in IPF patients. To identify the regulation of glutamine metabolism on the regeneration of AT2 cells during lung injury, RNA-seq and metabolic profiling were performed using AT2 cells from mice treated with bleomycin. Metabolic profiling showed that the levels of glutamine were decreased, and transcriptomic analysis showed that the gene expression levels of Gls1 and Gpt2 were downregulated. The top 30 most enriched GO terms of downregulated DEGs suggest that the glutamine catabolic process, Wnt signaling pathway, ATPase activity, and other cellular biological processes were altered during bleomycin injury. Notably, the transport of glutamine in AT2 cells of patients with IPF and mice with bleomycin-induced fibrosis was not exactly the same. In patients with IPF, AT2 cells showed a low level of glutamine exchange with both sides of the cell membrane. The type II membrane protein CD98 dimerizes with the light chains of transporter LAT1 to act as a chaperone and is located on the plasma membrane [41]. The ASCT2 sodium-dependent reverse transporter exchanges neutral amino acids with glutamine [42]. Reduced expression of SLC1A5, SLC7A5, and SLC3A2 indicates reduced glutamine uptake and efflux. However, the expression of these genes was stable during bleomycin-induced fibrosis in mice. This may result from the limitations of the bleomycin-induced pulmonary fibrosis mouse model. The pathological manifestations of lung tissues in mice 14 days after bleomycin administration were consistent with those of patients with IPF, but pulmonary fibrosis in mice during this period was in the progressive stage of the disease, whereas most patients with IPF in our single-cell data were probably in the terminal stage of the disease. Patients with end-stage IPF experience long-term nutrient depletion, and the efficiency of glutamine supply and utilization in cells is reduced.

Under inflammatory conditions, glutamine acts as an energy source for intestinal epithelial fuel substrate [43]. Glutamine treatment attenuates inflammation and endotoxin release in acute respiratory distress syndrome (ARDS) [44]. For SARS-CoV-2, Glutamine metabolic reprogramming is a possible approach to inhibit the pathogenesis of the virus through the regulation of GLS1, phosphoserine aminotransferase 1 (PSAT1), hypoxia-inducible factor-1α (HIF-1α), mTOR complex 1 (mTORC1), fructose-6-phosphate aminotransferase (GFAT), and Myc [45]. A recently published study found that media rich in glutamine supports intracellular metabolite production, including a variety of mitochondrial respiration intermediates and glycolysis intermediates, whereas DNA is more resistant to bleomycin damage and thus reduces cell death. It is possible that glutamine, rather than glucose, helps protect the lung epithelium from damage [46]. Glutamine metabolism is required to promote amino acid biosynthesis. Glutamine metabolism has been shown to increase the cellular concentrations of glycine and proline to promote amino acid biosynthesis for collagen production in myofibroblasts [47]. The glutamate-consuming enzymes phosphoserine aminotransferase 1 (PSAT1) produces 3-phosphoserine, an intermediate of the de novo serine and glycine synthesis pathway. In vitro, AOA can potentially inhibit PSAT1 and not only GOT2 and GPT2. In epithelial cells, the available evidence suggests that glutamine metabolism primarily provides an intermediate for mitochondrial oxidation [46]. This may explain why we found a decrease in the conversion of glutamine to TCA cycle intermediates in the mitochondria, whereas other cytoplasmic destinations of glutamine, such as participation in nucleotides, glycosylation, and amino acid synthesis, showed no changes in single-cell data from patients with IPF or in metabolomic data from mice with bl-induced lung fibrosis. Otherwise, the maintenance of regeneration function in AT2 cells requires the synergistic action of multiple metabolic pathways in vivo, and the metabolism of glutamine in the mitochondria is the only energy source for AT2 cells. Our previous study also reported the effects of glucose metabolism and lipid metabolism reprogramming on alveolar progenitor cell regeneration. Because of the relatively small number of alveolar progenitor cells used in our study, which were all primary mouse cells, we could not quantify mitochondrial respiration in AT2 cells directly using seahorse. Our study on glutamine metabolism is limited to the production of α-ketoglutaric acid, and we will investigate the role of α-KG supplementation in vitro in the repair of alveolar epithelium during fibrosis in the future.

## 5. Conclusions

This study highlights the role of glutamine metabolism in the regulation of alveolar epithelial regeneration in lung fibrosis, and the results obtained provide new ideas for the effective treatment of pulmonary fibrosis from the perspective of tissue regeneration. Additionally, the findings also highlight a new target for IPF-related clinical therapies and drug development.

## Data Availability

Not applicable.

## Supplementary Materials

The following are available online at https://www.mdpi.com/article/10.3390/biom12050728/s1, Figure S1: Reduced proliferation of AT2 cells during bleomycin-induced lung fibrosis in mice, Figure S2: Transcriptomic and metabonomic profiling, Figure S3: Schematic diagram of glutamine metabolism in AT2 cells from patients with IPF and from mice with bleo-17 mycin-induced lung fibrosis, Table S1: Detailed information of differential genes in Figure 1C.
