# Supplementary material for: Glutamine Metabolism Is Required for Alveolar Regeneration during Lung Injury
Source: Biomolecules. 2022 May 22;12(5):728. doi: 10.3390/biom12050728 (PMC9138637; doi:10.3390/biom12050728)
Supplement: Supplementary file 1 [file biomolecules-12-00728-s001.zip › biomolecules-1697582-Supplementary.pdf]

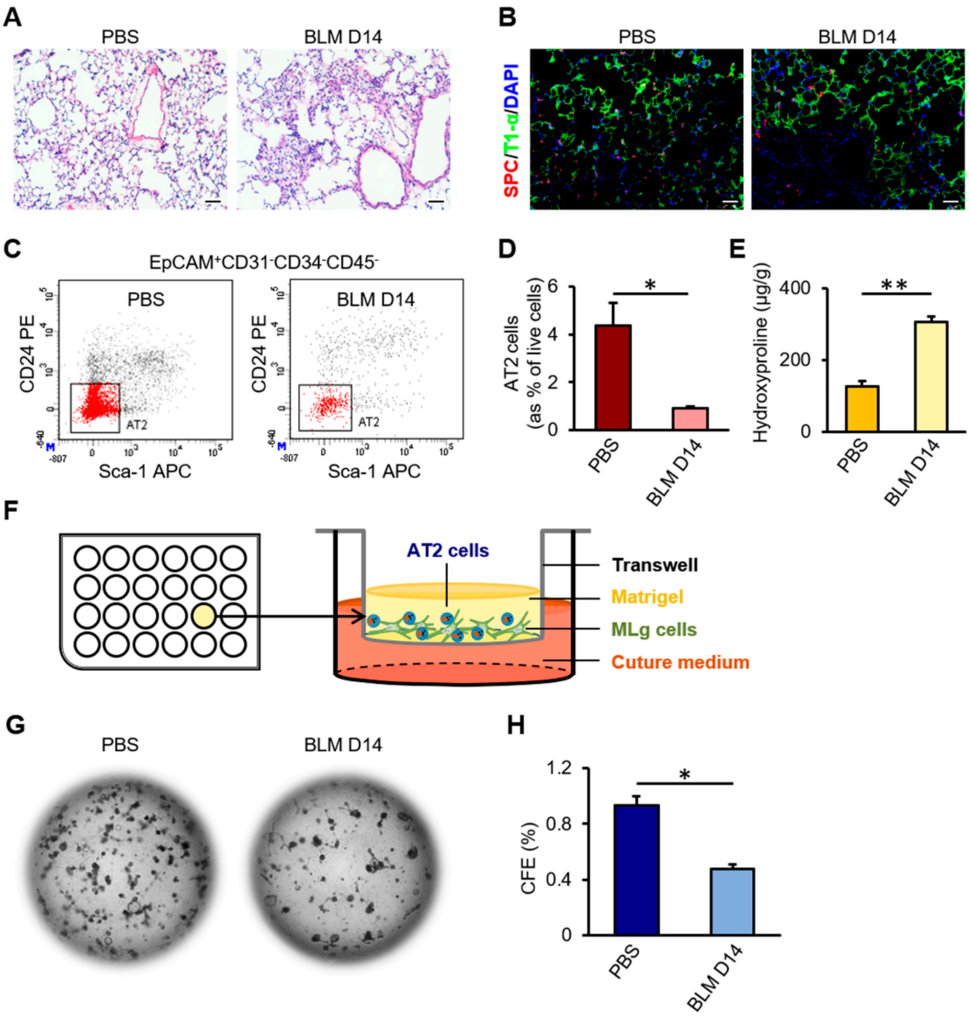

Figure S1. Reduced proliferation of AT2 cells during bleomycin-induced lung fibrosis in mice. (A) H&E staining of lung sections from mice with bleomycin injury. (B) Representative images of lung tissue stained with SPC (red), T1α (green), and DAPI (blue) after bleomycin injury. (C-D) Representative charts of flow cytometric analysis (Figure 1C) and a quantitative fractions of the abundance of survived AT2 cells (Figure 1D) in lungs of mice 14 days after bleomycin injury (n = 5). (E) The level of hydroxyproline in lungs of mice 14 days after bleomycin injury (n = 5). (F) Schematic illustration of organoid culture in vitro. (G) Representative images of organoid cultures at day 10 after AT2 cells seeding isolated from mice after bleomycin administration. (H) CFEs of organoid colonies of AT2 cells. *$p < 0.05$. Scale bars: 50 μm.

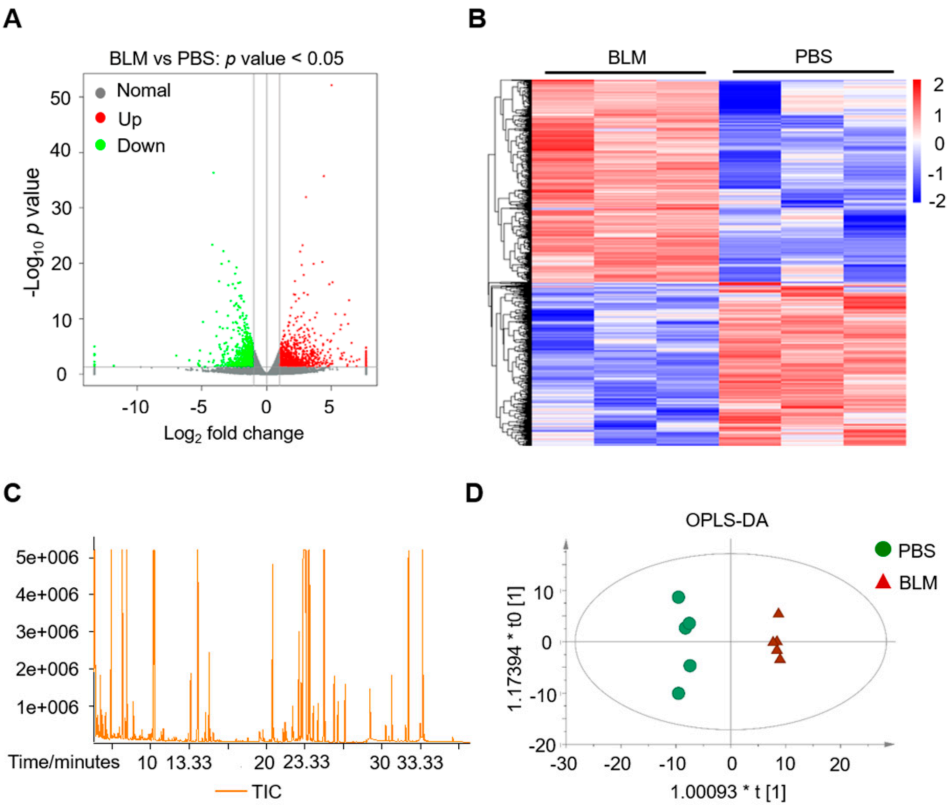

Figure S2. Transcriptomic and metabonomic profiling. (A) The volcano map presents the distribution of differences in gene expression levels between the BLM and PBS groups. Grey indicates the non-significantly differentially expressed genes. Red and green indicate upregulated and downregulated genes. (B) Heatmap showing transcriptional profiling of mouse AT2 cells isolated from control (n = 3) and BLM-challenged mice (n = 3) at day 14 (p value < 0.05, fold-change ≥ 2). (C) Total ion Current (TIC) of metabolites. (D) OPLS-DA plot of the metabolomics data in AT2 cells isolated from mice after BLM or PBS treatment (n = 5).

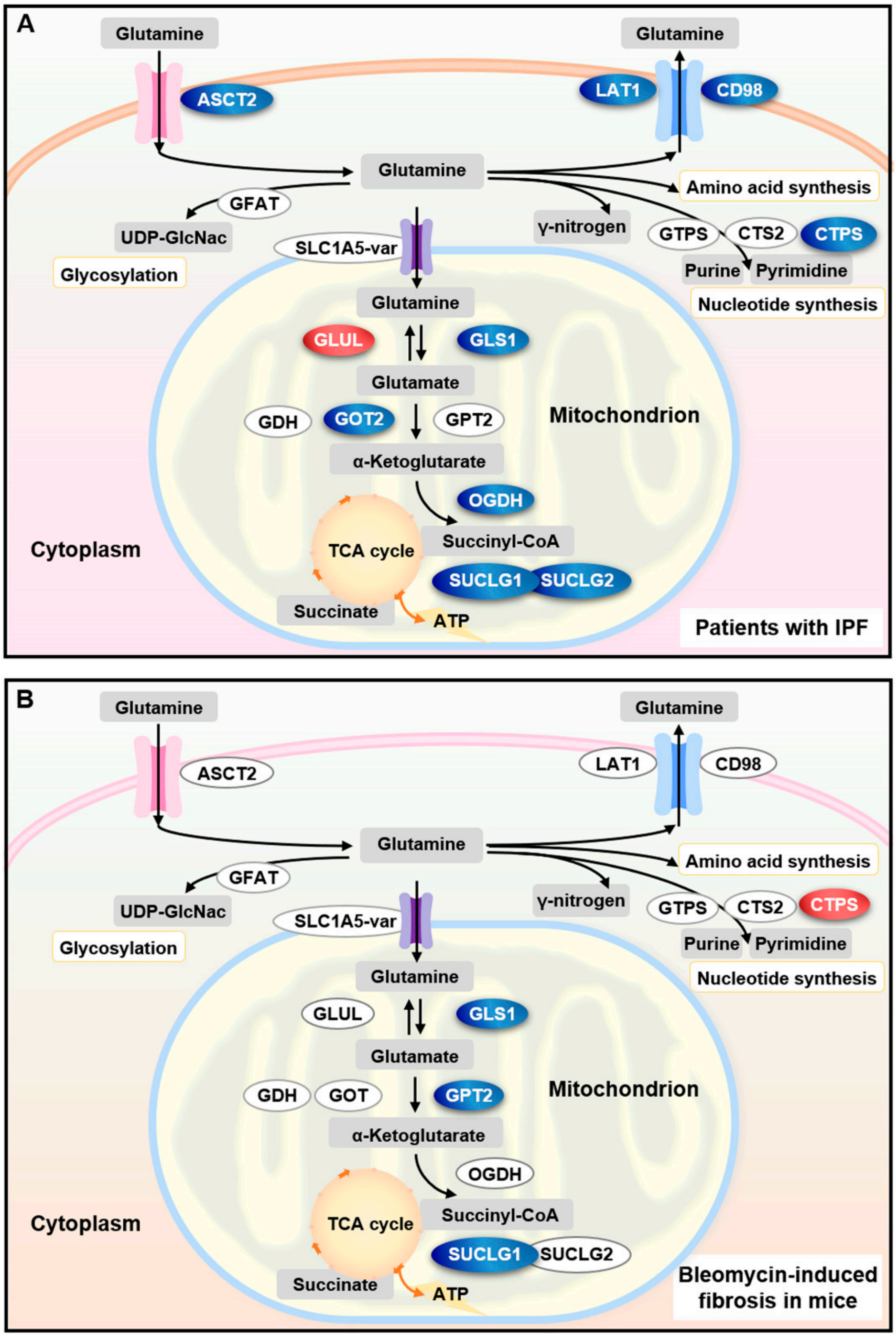

Figure S3. Schematic diagram of glutamine metabolism in AT2 cells from patients with IPF and from mice with bleomycin-induced lung fibrosis. In patients with IPF (A) and bleomycin fibrotic mice (B), glutamine enters into cells through the sodium-dependent reverse transporter SLC1A5 and SLC3A2/SLC7A5 heterodimers exchange glutamine into essential amino acids. In the cytoplasm, glutamine provides γ-nitrogen for nucleotides and hexosamine, and participates in the production of amino acids. After translocation into mitochondria by a SLC1A5 variant, glutamine is catalyzed by GLS1 to glutamate, which is converted by GDH or GOT or GPT2 to α-ketoglutarate which further produces ATP and supplements TCA cycle intermediates. When fibrosis occurs, glutamine metabolism in mitochondria decreases, and the weakening of glutamine exchange inside and outside AT2 cells is observed in patients with IPF.

Table S1. Detailed information of differential genes in Figure 1C.

Gene	p_val	avg_log2FC	Pct.1	Pct.2	p_val_adj
SFTPC	1.29E-75	-0.3441772	0.849	0.948	4.27E-71
ABCA3	2.68E-42	-0.1406377	0.644	0.893	8.88E-38
GLUL	1.68E-34	0.002505252	0.425	0.626	5.56E-30
GLS1	9.17E-54	-0.37216021	0.407	0.625	3.03E-49
GOT2	4.45E-45	-0.017115976	0.109	0.22	1.47E-40
OGDH	2.61E-60	-0.090757489	0.204	0.377	8.64E-56
SUCLG1	2.93E-110	-0.144963592	0.231	0.479	9.69E-106
SUCLG1	4.44E-94	-0.176985154	0.232	0.457	1.47E-89
